# Histopathological Observation of Immunized Rhesus Macaques with Plague Vaccines after Subcutaneous Infection of *Yersinia pestis*

**DOI:** 10.1371/journal.pone.0019260

**Published:** 2011-04-29

**Authors:** Guang Tian, Yefeng Qiu, Zhizhen Qi, Xiaohong Wu, Qingwen Zhang, Yujing Bi, Yonghai Yang, Yuchuan Li, Xiaoyan Yang, Youquan Xin, Cunxiang Li, Baizhong Cui, Zuyun Wang, Hu Wang, Ruifu Yang, Xiaoyi Wang

**Affiliations:** 1 State Key Laboratory of Pathogen and Biosecurity, Beijing Institute of Microbiology and Epidemiology, Beijing, China; 2 Laboratory Animal Center, Academy of Military Medical Science, Beijing, China; 3 Qinghai Institute for Endemic Disease Prevention and Control of Qinghai Province, Xining, China; University of Pittsburgh, United States of America

## Abstract

In our previous study, complete protection was observed in Chinese-origin rhesus macaques immunized with SV1 (20 µg F1 and 10 µg rV270) and SV2 (200 µg F1 and 100 µg rV270) subunit vaccines and with EV76 live attenuated vaccine against subcutaneous challenge with 6×10^6^ CFU of *Y. pestis*. In the present study, we investigated whether the vaccines can effectively protect immunized animals from any pathologic changes using histological and immunohistochemical techniques. In addition, the glomerular basement membranes (GBMs) of the immunized animals and control animals were checked by electron microscopy. The results show no signs of histopathological lesions in the lungs, livers, kidneys, lymph nodes, spleens and hearts of the immunized animals at Day 14 after the challenge, whereas pathological alterations were seen in the corresponding tissues of the control animals. Giemsa staining, ultrastructural examination, and immunohistochemical staining revealed bacteria in some of the organs of the control animals, whereas no bacterium was observed among the immunized animals. Ultrastructural observation revealed that no glomerular immune deposits on the GBM. These observations suggest that the vaccines can effectively protect animals from any pathologic changes and eliminate *Y. pestis* from the immunized animals. The control animals died from multi-organ lesions specifically caused by the *Y. pestis* infection. We also found that subcutaneous infection of animals with *Y. pestis* results in bubonic plague, followed by pneumonic and septicemic plagues. The histopathologic features of plague in rhesus macaques closely resemble those of rodent and human plagues. Thus, Chinese-origin rhesus macaques serve as useful models in studying *Y. pestis* pathogenesis, host response and the efficacy of new medical countermeasures against plague.

## Introduction

Plague is a zoonotic infectious disease caused by the Gram-negative bacterium *Yersinia pestis*. Historically, plague is an awful infectious disease that afflicts human populations, leading to millions of deaths. Plague has been recently classified as a re-emerging disease by the World Health Organization [1] and has attracted considerable attention because of its potential misuse as an agent of biological warfare or bioterrorism [2]. Most human plague cases clinically present as one of three primary forms—bubonic, septicemic and pneumonic. Bubonic plague is the most common form of the disease, which occurs when *Y. pestis* is inoculated into the skin. Primary bubonic or septicemic plague is usually caused by fleabite among rodent reservoir hosts and most human cases are usually transmitted from an infected rodent via the bite of an infected flea, whereas pneumonic plague usually spread by respiratory droplets. Patients with primary bubonic plague can develop secondary septic or pneumonic infection, which can then be spread from person-to-person via respiratory droplets generated from sneezing and coughing [3].

To evaluate plague vaccines, several animal models, such as mice, guinea pigs, rabbits [4] and cynomolgus macaques [5], have been used to determine protective efficacy against *Y. pestis* challenge and antibody responses to plague vaccines. On the other hand, to assess the animal models of bubonic plague, pathological characteristics during bubonic infection have been investigated in mice [6], cats [7] and guinea pigs [3]. Pneumonic plague is the form most likely to be observed in biological warfare or bioterrorism events and interest in animal models has been raised to support plague therapeutic and vaccine studies. Pathological features of pneumonic plague have been observed in mice [8], [9], rats [10], Indonesian cynomolgus macaques [11] and African green monkeys [12].

Protective efficacy is usually evaluated in terms of antibody titers and survival rate after challenging with *Y. pestis* [5]. In our previous study [13], Chinese-origin rhesus macaques were used as an animal model to evaluate the protective efficacy of subunit vaccines SV1 (20 µg F1 and 10 µg rV270) and SV2 (200 µg F1+100 µg rV270), and a live attenuated vaccine EV76 against subcutaneous challenge with a virulent *Y. pestis* 141 strain. Complete protection was observed in the animals immunized with SV1, SV2, and live attenuated vaccine EV76 against subcutaneous challenge with 6×10^6^ colony-forming units (CFU) of virulent *Y. pestis* strain 141. The control animals succumbed to the same dose of *Y. pestis* 141 within 3 to 5 days [13]. However, whether F1+rV270 subunit vaccines and EV76 live attenuated vaccine can effectively protect the immunized animals from any pathological changes remain unknown.

In the present study, we examined liver, spleen, lung, kidney, heart and lymph node tissues from Chinese-origin rhesus macaques immunized with SV1, SV2, and EV76 that were subcutaneously infected with virulent *Y. pestis* 141. Additionally, the control animals were examined by histopathological methods. The *Y. pestis* distribution in tissues was determined with Giemsa staining under light microscopy, transmission electron microscopy, and immunohistochemistry. In addition, the glomerular immune deposits in the immunized animals and in the control animals were checked by electron microscopy.

## Results

### Tissue injury after infection with *Y. pestis*

**Figure 1:**
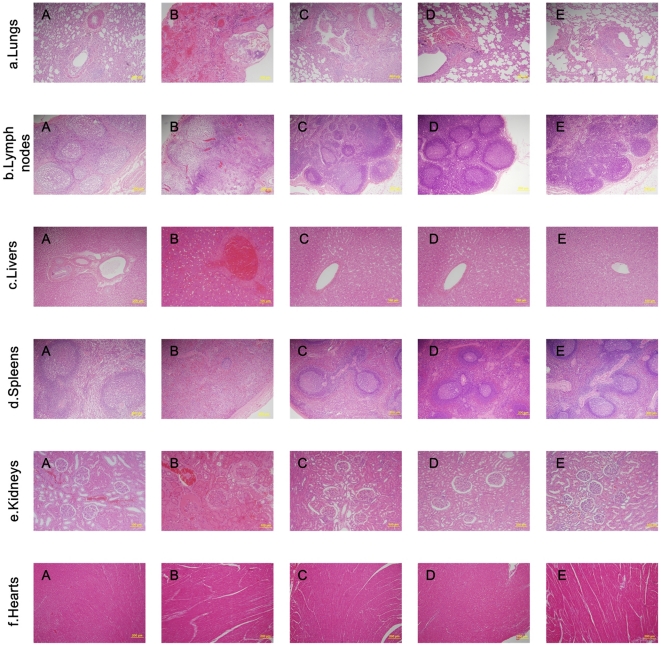
Histopathology of the organs from the immunized animals, the control animals, and the naïve control animal. Tissue sections were stained with hematoxylin and eosin for pathological examination after infection with *Y. pestis*. (a) Tissue sections from the naïve control animal that was neither immunized with plague vaccines or aluminum hydroxide adjuvant nor infected with *Y. pestis*. (b) Tissue sections from the control animal that only received aluminum hydroxide adjuvant before it was infected by virulent *Y. pestis* strain 141. (c) Tissue sections from the animals immunized with SV1 and then infected with virulent *Y. pestis* strain 141. (d) Tissue sections from the animals immunized with SV2 and then infected with virulent *Y. pestis* strain 141. (e) Tissue sections from the animals immunized with EV76 and then infected with virulent *Y. pestis* strain 141.

After challenging with virulent *Y. pestis* 141, the lungs, livers, kidneys, lymph nodes, spleens, and hearts of the animals immunized with SV1, SV2, and EV76, and the control animals immunized with adjuvant were collected, fixed in 10% neutral buffered formalin, and prepared for HE staining. One normal macaque that was neither immunized with plague vaccines or adjuvant nor infected with *Y. pestis* was used as the naïve control. Compared with the lung, lymph node, liver, spleen, kidney, and heart tissues of the naïve control animal (Fig. 1 a–f, Panel A), no changes in histopathology were found in all examined tissues from the animals immunized with SV1 (Fig. 1 a–f, Panel C), SV2 (Fig. 1 a–f, Panel D), and EV76 (Fig. 1 a–f, Panel E), whereas the control animals showed evident alterations in the lungs, lymph nodes, livers, spleens and kidneys (Fig. 1 a–e, Panel b). Haemorrhage, effusion and edema, inflammatory cell infiltration, and abscess containing *Y. pestis* were observed in the lung tissues of the control animals (Fig. 1 a, Panel B). Disappearance of recognizable architecture, reduced number of lymphocytes, severe congestion and edema were observed in the lymph nodes (Fig. 1 b, Panel B). The liver tissues of the control animals showed hepatocyte swelling, vacuolar degeneration, dilatation and hyperraemia of the central vein of hepatic lobules, and slight congestion within sinus hepaticus (Fig. 1 c, Panel C). The spleen tissues had reduced amount of white pulp, acinus lienalis, and lymphocytes and displayed splenic cord edema (Fig. 1 d, Panel B). Acinus renis analosis, renal capsule effusion, interstitial edema, and vascular engorgement were observed in the kidney tissues of the control animals (Fig. 1 e, Panel B). There were no evident changes in the heart tissues of the control animal (Fig. 1 f, Panel B).

### Localization of *Y. pestis* within infected animal organs

**Figure 2:**
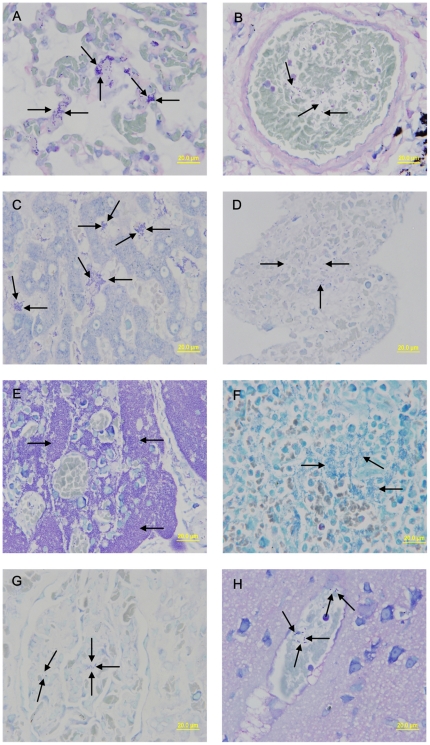
Giemsa staining was done to visualize the bacteria in tissues. The bacteria were clearly visible within the tissues from the lungs, livers, kidneys, lymph nodes, brains, spleens, and blood from heart of the control animals that were only immunized with aluminum hydroxide adjuvant before they were infected with virulent *Y. pestis* strain 141. The lungs showed several large clumps of bacteria in the alveolar septum (a, arrows). Bacteria were also found inside blood vessels in the lungs (b, arrows) and blood from heart (d, arrows). Several large clumps of bacteria were shown in the hepatic sinus (c, arrows). Medullary cords of the lymph nodes were damaged and occupied by a large number of bacteria (e, arrows). The spleen show many large clumps of bacteria (f, arrows). Smaller clumps of bacteria are shown within the glomcrulus (g, arrows) and blood vessels in brain tissue (h, arrows).

Giemsa staining was used to identify the presence of *Y. pestis* (based on size and morphology) in the different organs of the immunized animals, as well as those of the control animals after infection with virulent *Y. pestis* strain 141. No bacteria were observed within the tissues of lungs, livers, kidneys, lymph nodes, brains, spleens, and blood from heart of the immunized animals, whereas bacteria were clearly visible within those of the control animals (Fig. 2, Panels A–H).

**Figure 3:**
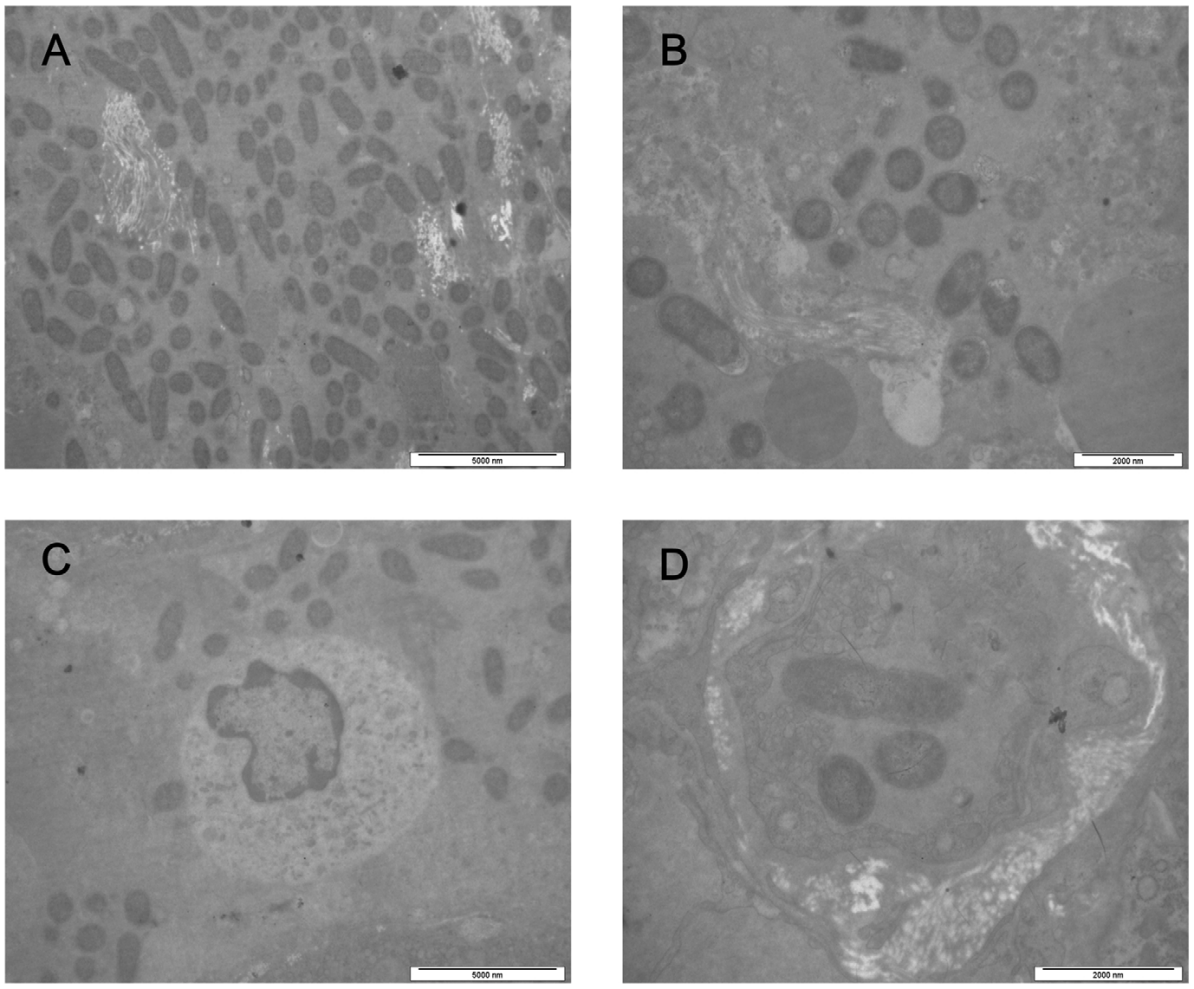
Transmission electron micrographs of the organs of the control animals. Transmission electron micrographs of the lymph nodes, spleen, liver, and lungs of the control animals that were only immunized with aluminum hydroxide adjuvant and then infected with virulent *Y. pestis* strain 141. The tissues were fixed in 3.1% glutaraldehyde solution and then postfixed in 1% osmium tetroxide. Afterwards, the tissue samples were dehydrated with a serial alcohol gradient, and then embedded in Epon812. Ultrathin sections were stained with uranyl acetate and lead citrate and examined under a Philips Tecnai 10 transmission electron microscope. Ultrastructural examination shows that lymph node tissue structures were destroyed and a large number of bacteria are found in the interstitium (a). The Splenic pulp shows a large number of bacteria and its structure was destroyed (b). A large number of bacteria are found around Kupffer's cells (c). Three *Y. pestis* were observed in blood vessel of lungs (d).

Ultrastructural examination also revealed bacteria in the lymph nodes, spleens, livers and lungs of the control animals (Fig. 3, Panels A–D). No bacteria were observed in the corresponding organs of the immunized animals.

**Figure 4:**
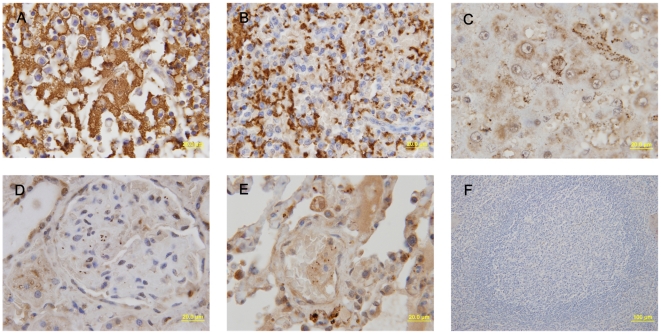
The F1 antigen of *Y. pestis* was identified by Immunohistochemitry staining. The F1 antigen of *Y. pestis* was identified by Immunohistochemitry staining in formalin-fixed paraffin-embedded tissues from the control animals that were only immunized with aluminum hydroxide adjuvant and then infected with virulent *Y. pestis* strain 141. The sections were incubated for 12 h with the purified rabbit anti-F1 antigen of *Y. pestis* polyclonal antibody at 4°C, and incubated for 10–20 min with the polyperoxidase-anti-rabbit IgG at 37°C. The slides were stained with 3, 3′-diaminobenzidine tetrahydrochloride (DAB). Finally, the sections were rinsed, counterstained, dehydrated, cleaned, mounted and examined by light microscopy. Bacteria were visualized as those expressing F1 antigen (brown and yellow stain). Numerous bacteria were observed in the tissues of the lymph node (a), spleen (b), liver (c), kidneys (d) or lungs (e). No bacterium was found in the control spleen (f).

The F1 antigen of *Y. pestis* was identified by immunohistochemitry staining in the formalin-fixed paraffin-embedded tissues. Bacteria could be visualized as those expressing the F1 antigen (brown and yellow stain). Numerous bacteria were observed in the tissues of the control animals (Fig. 4. Panels A–E) and no bacteria were found in the naïve control animal tissues and in tissues of the immunized animals.

### Inspection of basement membranes

**Figure 5:**
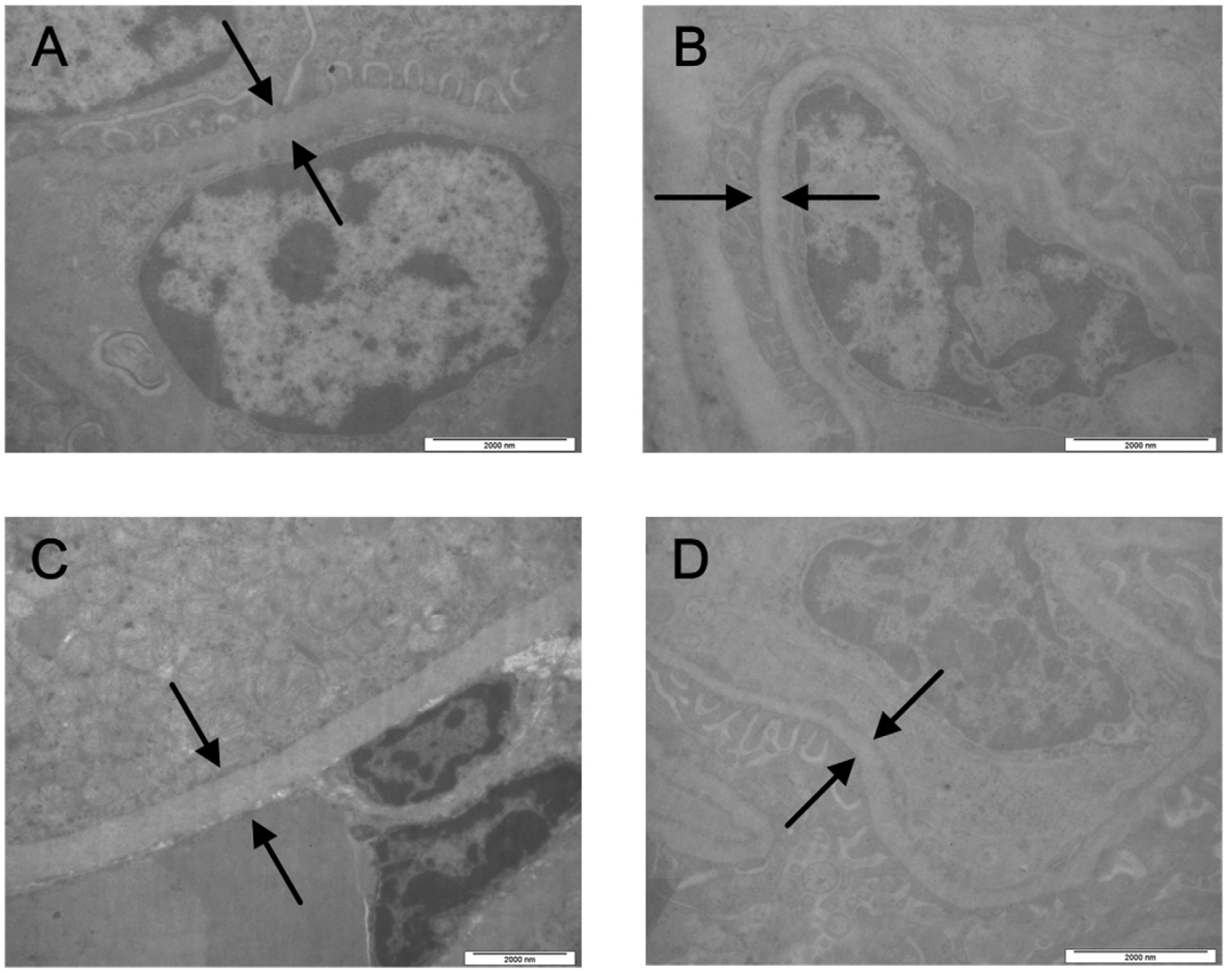
Ultrastructure of the glomerular basement membranes of the immunized animals after the *Y. pestis* challenge. The control animals infected with *Y. pestis* were observed under a transmission electron microscope. Transmission electron micrograph of the glomerular basement membrane (GBM) of the control animal that that were only immunized with aluminum hydroxide adjuvant and then infected with virulent *Y. pestis* strain 141 (a), the animal immunized with EV76 (b), the animal immunized with subunit vaccine SV1 (c), and the animal immunized with subunit vaccine SV2 (d).

The ultrastructure of the GBMs in the immunized and the control animals after the *Y. pestis* challenge were observed under transmission electron microscopy. Histological examination of renal tissues revealed no signs of immune complex deposition on the GBMs (Fig. 5, Panels A–D).

## Discussion

Currently, evaluation of plague vaccines is often based on antibody response, cell-mediated immunity and protective efficacy. Subunit vaccines in alhydrogel elicit a predominant response to the F1 and V antigens by procucing IgG1, and total IgG titres correlate with protection against plague in mice [14]. However, specific IgG titers to F1 and V antigens do not correlate with protective efficacy in non-human primates [15], [16]. A great deal of evidence has demonstrated that antibody titers alone do not suffice in predicting the protective efficacy of plague vaccines. Vaccines that elicit both humoral and cellular immunity can contribute to optimal defense against pneumonic plague [17]. However, our previous study [4] and several reports [18], [19], [20] have demonstrated that subunit vaccines in alhydrogel elicit robust humoral immunity, but their potential in priming effective cellular immunity has yet to be demonstrated. Under these circumstances, mortality in response to experimental plague challenges is the most important data for evaluating the efficacy of plague subunit vaccines. Although complete protection against subcutaneous challenge with 6×10^6^ CFU of virulent *Y. pestis* strain 141 was observed in the Chinese-origin rhesus macaques immunized with SV1, SV2, and EV76 and the control animals succumbed to the same dose within 3 to 5 days [13], we are unsure whether the vaccines can effectively protect the immunized animals from any pathological changes in different organs, whether the control animals died from specific *Y. pestis* infection, or whether Chinese-origin rhesus macaques subcutaneously infected with virulent *Y. pestis* has similar histopathologic features as human bubonic plague or those of other animals.

Cornelius et al. examined the lungs of infected cynomolgus macaques by aerosol with *Y. pestis* CO92 for histopathologic changes associated with infection. The results showed clear differences between the adjuvant-immunized animals and the cynomolgus macaques immunized with rLcrV, rV10, or rF1. The lungs of all the control animals and one rF1-vaccinated macaque that succumbed to infection had multiple areas of severe congestion and hemorrhage, whereas other immunized macaques did not demonstrate any clear gross or histopathological changes in their lung tissues [5]. To evaluate histopathologically the protective efficacy of the F1+rV270 subunit vaccine and the EV76 control vaccine, the lung, lymph node, liver, spleen, kidney, and heart tissues from the immunized and control macaques were examined under microscopy. The immunized animals did not display any histopathological lesions in all the examined tissues, whereas the control animals showed different degrees of pathological alterations in the lungs, lymph nodes, livers, spleens, and kidneys. In addition, Giemsa staining, ultrastructural examination, and immunohistochemical staining revealed bacteria in all the organs of the control animals, whereas no bacteria was observed in the immunized animals. However, in the present study, the tissues were only examined at 14 days after the challenge. The possibility of histopathological lesion and bacterial presence at earlier stages of infection should not be excluded. In our previous study, post-mortem analysis of the *Y. pestis* load in the livers, spleens, lungs, lymph nodes, and blood from hearts of macaques immunized with subunit vaccines (SV1 and SV2) and live attenuated vaccine EV76 showed that *Y. pestis* was eliminated from the immunized animals, whereas *Y. pestis* was isolated from the organs of the control animals that died during the challenge [13]. Our previous post-mortem analysis and the current study demonstrat that SV and EV76 can effectively protect Chinese-origin rhesus macaques from pathological changes in the examined organs and eliminate *Y. pestis*. The control animals died from the lesions of many organs caused specifically by *Y. pestis* infection. Although survival rates in experimental infection are important for evaluating the efficacy of plague subunit vaccines, lesion severity may have better correlation with survival. Non-specific death is often observed in the course of experiments, so pathological observation can precisely provide the most important criterion for evaluating the efficacy of plague vaccines.

Histopathological examination is useful for evaluating the efficacy of new plague vaccines and for better understanding of the pathogenesis of the disease. The current scenario suggests that bacteria enter the lymphatic system from infected fleabite site, localize in the regional draining lymph node, and grow to large numbers, causing formation of “buboes” (swollen lymph nodes). Without timely effective treatment, *Y. pestis* spreads from the lymph nodes to the blood, followed by colonization of internal organs and secondary bacteremia. Patients usually die from uncontrolled septicemia, endotoxic shock, and disseminated intravascular coagulation [3], [10]. Although the pathological characteristics of pneumonic plague have been investigated in nonhuman primates, such as Indonesian cynomolgus macaques [11] and African green monkeys [12], the present study is the first to provide a thorough description of the histopathology of bubonic plague in Chinese-origin rhesus macaques. Although the chronological course of infection after *Y. pestis* inoculation into the skin of Chinese-origin rhesus macaques was not investigated, the results reveal that subcutaneous *Y. pestis* infection in Chinese-origin rhesus macaques results in bubonic, septicemic, and pneumonic plague. The histopathologic features of plagues closely resemble those of rodent and human bubonic plague [10]. Control animals develop a bubonic form of the disease with varying degrees of internal organ involvement (lungs, lymph nodes, liver, spleens, and kidneys) except for heart tissues. Giemsa staining revealed that *Y. pestis* escape the inoculation site and enter the blood stream, suggesting that bubonic plague progresses to septicemic plague in the course of 3–5 days. More importantly, there were clear signs of infection in the lungs of the control animals, indicating that subcutaneous injection resulted in secondary pneumonic infection. Bubonic plague, the most common form of the disease in rodents and humans, is usually acquired from the bite of an infected flea. In the present study, the subcutaneously infected Chinese-origin rhesus macaques developed bubonic plague, followed by pneumonic and septicemic plague. Thus, Chinese-origin rhesus macaques can serve as useful models for studying microbial pathogenesis, host response, and the efficacy of new medical countermeasures against plague.

Antigen clearance is normally dependent on the formation of antigen-antibody complexes and their removal by mononuclear phagocytes of the reticuloendothelial system. However, occasionally, immune complexes persist and eventually deposit in a range of tissues and organs. Under such abnormal circumstances, immune complexes are capable of triggering a wide variety of inflammatory processes by cell-mediated or complement-mediated events, causing damage to underlying tissues [21], [22]. Several studies have demonstrated that sequential administration of a cationic antigen and its corresponding antibodies or the initial administration of cationic antibodies followed by the specific antigen leads to formation of subepithelial immune deposits in the capillary basement membrane of glomeruli. Deposition requires vascular permeability and occurs mostly at sites of high pressure and turbulence such as the kidneys. In addition, immune complexes often deposit around the walls of small blood vessels. Immune complex deposition occurs mostly in glomerular capillaries, choroid plexus, and lung alveoli [21], [23], [24]. Plague vaccines elicit high level of antibodies specific to the F1 or V antigen in humans or animals. Repeated vaccination is often needed to maintain good efficacy, and occasionally vaccinated humans or animals are still subject to *Y. pestis* infection. Under these two circumstances, the formation of immune complex deposits in the glomerular capillary basement membrane may cause serious kidney damage. However, whether plague vaccines cause damage to the renal tissues of the immunized animals after *Y. pestis* infection has not been reported. In the present study, the GBMs of the immunized macaques were examined under electron microscopy to evaluate the safety of the subunit vaccines. The results show no signs of immune complex deposition on glomerular basement membranes. The current study is a follow-up research to our previous study [13], and does not include a positive control with respect to immune complex deposition on the GBMs. Therefore, we suggest that antibodies induced by SV1, SV2, and EV76 might not result in unintended effects that can potentially cause toxicity, such as an immune-mediated disease resulting from the deposition of immune complexes in Chinese-origin rhesus macaques.

In conclusion, we have demonstrated that plague subunit vaccines and live attenuated vaccine EV76 can protect rhesus macaques from any histopathologic lesion in the lungs, livers, kidneys, lymph nodes, spleens and hearts of the immunized animals. Pathological alterations were seen in the corresponding tissues of the control animals. Giemsa staining, ultrastructural examination, and immunohistochemical staining revealed bacteria in some organs of the control animals, whereas no bacteria were observed in the immunized animals. The results show that the plague vaccines can effectively protect rhesus macaques from pathological changes and eliminate *Y. pestis* from the immunized animals. The control animals died from multi-organ lesions specifically caused by the *Y. pestis* infection. These observations are consistent with the protective efficacy and antibody responses determined in our previous study [13]. Thus, histopathological observation, Giemsa staining, ultrastructural examination, and immunohistochemical staining provide effective means for evaluating plague vaccines.

## Materials and Methods

### Vaccines and animals

The subunit vaccine F1+rV270 was prepared as previously mentioned, which comprise native F1 and rV270 antigens adsorbed onto 25% (v/v) aluminum hydroxide adjuvant in PBS buffer [4]. Adsorption of the proteins onto the adjuvant was checked by subtracting the remaining protein in the supernate from the total amount of proteins added. The live attenuated vaccine EV76 was obtained from the Lanzhou Institute of Biological Products (LIBP), China. Adult male and female Chinese-origin rhesus macaques were obtained from the Laboratory Animal Center, Academy of Military Medical Science, China [under licensed from the Ministry of Health in General Logistics Department of Chinese People's Liberation Army, Permit No. SCXK-2007-004]. The animals were 3–6 years old and weighed between 3 and 6 kg. The animal experiments were conducted in strict compliance with animal welfare regulations, and the steps taken to ameliorate suffering were in accordance with the recommendations of the Weatherall Report on the use of non-human primates in research [25]. All of the animals were raised in an air-conditioned laboratory with an ambient temperature of 21–25°C, a relative humidity of 40%–60% and a 12 h light/dark cycle. Each animal was kept in a suspended stainless steel wire-bottomed cage and provided with a restricted diet of approximately 150 g of standard monkey keeping diet per day. Fresh fruit was given once daily to each of the animals, and water was available *ad libitum* during the entire course of this study.

### Animal immunizations

A total of 14 Chinese-origin rhesus macaques were divided into four groups, three experimental groups and one control group. Each of the three experimental groups contained four animals (two male and two female). The alum-immunized control group had two animals (one male and one female). The animals in each experimental group were intramuscularly injected in the forelimbs with one of the following vaccines: SV1 (20 µg of F1 and 10 µg of rV270), SV2 (200 µg of F1 and 100 µg of rV270), and EV76 [ one-half of the human dose (8×10^8^ cells)]. Each animal in the control group was intramuscularly given 25% aluminum hydroxide adjuvant only. At 21 days after the first immunization, all the animals were boosted with an identical dose at the same injection sites.

### Animal Infections

The virulent *Y. pestis* strain 141 was isolated from *Marmota himalayana* in the Qinghai Tibet Plateau and has a median lethal dose (MLD) of 5.6 CFU for BALB/c mice, and 17.8 CFU for guinea pigs and New Zealand white rabbits by subcutaneous route. The *Y. pestis* was cultured in Luria broth at 28°C for 18 h, quantified by Maxwell turbidimetry, and diluted in sterile phosphate-buffered saline (PBS). The number of *Y. pestis* in the dilution was verified by colony-forming units (CFU) on *Y. pestis* selective agar medium. The 14 immunized animals were infected subcutaneously with 6×10^6^ CFU on Week 10 after the primary immunization and closely observed for 14 days. All the animal experiments were performed in ABSL-3 laboratory.

### Histopathology

Tissues collected from dead animals were placed into 10% neutral buffered formalin, dehydrated through a serial alcohol gradient (70%, 80%, 90%, 95%, and 100%), cleared with xylene, infiltrated with wax, and then embedded in paraffin [26]. The surviving animals at Day 14 after the challenge and one uninfected animal were humanely killed by intraperitoneal injection of barbital sodium. As stated above, their tissues were removed and fixed in 10% neutral buffered formalin for paraffin block preparation. Tissue sections were stained with hematoxylin and eosin (HE) for histopathological examination, and the presence of *Y. pestis* was detected by Giemsa staining under light microscopy.

### Ultra-structural observation

A small section of tissues was removed, fixed for 6 h in 3.1% glutaraldehyde solution at 4°C and then post-fixed for 2 h in 1% osmium tetroxide at 4°C. Afterwards, the tissue samples were dehydrated using a series of alcohol gradient (50%, 70%, 90%, and 100%), and then embedded in Epon 812. Ultrathin sections were mounted on 230-mesh copper grids, and then stained with 1% uranyl acetate and lead citrate, and finally examined for bacteria and immune complex deposition on the glomerular basement membranes (GBMs) under a Philips Tecnai 10 transmission electron microscope [27].

### Immunohistochemistry (IHC)

IHC staining was performed following the user's manual of the PV-9000 Kit (ZSGB-Bio) [28]. Briefly, after the paraffin-embedded tissue sections were deparaffinized and rehydrated, the sections were subjected to antigen exposure in citrate buffer solution (0.1 M, pH 6.0) by microwaving at 95°C for 20 min, and incubated with 3% H_2_O_2_ in methanol for 10 min to block endogenous peroxidase activity. The sections were incubated for 12 h with the purified rabbit anti-F1 antigen of *Y. pestis* polyclonal antibody at 4°C, whereas the control spleen tissues were incubated in PBS. The sections were incubated with Polymer Helper for 20 min at 37°C, and then with polyperoxidase antirabbit IgG (ZSGB-Bio) for 10–20 min at 37°C. The slides were stained with 3, 3′-diaminobenzidine tetrahydrochloride (DAB). Finally, the sections were rinsed, counterstained, dehydrated, cleaned, mounted, and examined under light microscopy [29].
